# Prognostic value of the immunohistochemistry correlation of Ki-67 and p53 in squamous cell carcinomas of the larynx

**DOI:** 10.1016/S1808-8694(15)30145-2

**Published:** 2015-10-18

**Authors:** Ricardo Boose Rodrigues, Rafael da Ros Motta, Simone Márcia dos Santos Machado, Eduardo Cambruzzi, Eduardo Walker Zettler, Claudio Galleano Zettler, Geraldo Pereira Jotz

**Affiliations:** a Specialist in Pathology, MD, Pathologist, Pathology Service at Universidade Luterana do Brasil; b Resident Pathologist at Universidade Luterana do Brasil; c PhD student in the Pathology Graduate Program at FFFCMPA, Supervisor at the Medical Residency in Pathology at Universidade Luterana do Brasil; d PhD in Pathology at FFFCMPA, Adjunct Professor of Pathology in the Medicine Program at Universidade Luterana do Brasil; e PhD in Medicine at UFRGS, Adjunct Professor of Pneumology in the Medicine Program at Universidade Luterana do Brasil; f PhD in Pathology at FFFCMPA, Adjunct Professor of Pathology and Head of the Pathology Service at Universidade Luterana do Brasil; g PhD in Otorhinolaryngology, Head and Neck Surgery at UNIFESP. Post-PhD in Otorhinolaryngology at Pittsburgh University, Adjunct Professor and Head of the Otorhinolaryngology, Head and Neck Surgery Service at Universidade Luterana do Brasil. Adjunct Professor at the Department of Morphologic Sciences at UFRGS. Pathology Service - Universidade Luterana do Brasil

**Keywords:** squamous cell carcinoma, immunohistochemistry, larynx, p53, prognostic

## Abstract

Prognostic histological factors may contribute to determine the evolution of this neoplasia. **Aim**: To correlate p53 and Ki-67 immunohistochemical expression with age, histological degree, lymph node involvement and pathological staging in patients with laryngeal epidermoid carcinomas. **Methods**: We assessed thirty consecutive cases of laryngeal epidermoid carcinomas submitted to immunohistochemistry to check the expression of p53 e Ki-67 antibodies. **Results**: Mean age was of 56.2 years and the immunoexpression of the markers was observed in the group with more than 50 years of age, especially that o the ki-67 antibody (p=0.032). There was no relation between p53 and Ki-67 with lymph node involvement. Ki-67 was expressed in 70% of the high histology level cases and in 80% in the low histology ones; while p53 was of 70% only in the high level cases. Pathology staging showed that in the group of advanced carcinomas, p53 expression was of 61.5%, while Ki-67 proved positive for the early cases (100%) and advanced (73.1%). **Conclusion**: There were no significant differences between p53 and Ki-67 immunoexpression in laryngeal epidermoid carcinoma, except in the group of patients with more than 50 years of age, when Ki-67 expression was significantly higher.

## INTRODUCTION

Laryngeal cancer accounts for approximately 12,000 new cases per year in the United States and for 2% of all cancer-related deaths^1^. Some 130,000 new cases of laryngeal cancer are recorded in the world, affecting predominantly males at a rate of 7:1. Laryngeal tumors are among the most frequent cases of head and neck cancer, as they account for approximately 25% of the malignant tumors involving this area. About 2/3 of these tumors appear in the glottis and 1/3 involve the supraglottal region.

Epidermoid carcinomas are the most common histological type, including keratinizing (well-differentiated) and non-keratinizing tumors. They account for 2.2% of malignant neoplasms in men and 0.4% in women^2^, ^3^. Laryngeal carcinomas may involve all three anatomical sites, namely the glottis, subglottis, and supraglottal region.

The following histological prognostic factors for laryngeal carcinomas are considered: tumor site, histological type, histological grade, and lymph node status. Lymph node involvement is one of the most important prognostic factors for laryngeal carcinomas.

Cell growth suppressor genes and cell death regulator genes are relevant variables in tumor evolution; among them is gene p534-6. Gene p53 is a proto-oncogene that regulates cell growth. It singles out cells with DNA alterations, interrupts the growth cycle on stage G1, and places altered cells on G0. It then promotes cell repair to send the cell back to the cycle or, if that cannot be done, promotes cell death. The correlation between p53 expression and prognosis for various types of cancer has been well demonstrated^7^. The immunohistochemical expression of p53 leads to increased protein expression or mutation.

Cell proliferation is also seen as a fundamental biologic mechanism in oncogenesis^8^ and in the detection of cell growth fraction markers such as antibody Ki-67, used as a prognostic factor in a wide variety of tumors^9^, ^10^, ^11^.

Correlation between high proliferative activity and worse prognosis for laryngeal and head and neck tumors has shown, particularly for neoplasms invading structures located adjacently to the tumor”s site of origin^4^, ^5^, ^8^, ^9^. This study aims to relate the immunohistochemical expression of gene p53 and antibody Ki-67 to established prognostic factors (age, histological grade, lymph node status, and disease staging) in patients with laryngeal epidermoid carcinoma.

## MATERIALS AND METHOD

This study comprises a review of thirty consecutive cases of laryngeal epidermoid carcinoma diagnosed at the Head and Neck Service at Hospital Luterano da Universidade Luterana do Brasil between January of 2004 and September of 2006. All patients underwent laryngectomy and neck clearance. Our sample includes all cases of epidermoid carcinoma, regardless of tumor location. The cases were examined by the Pathology Service using conventional hematoxylin-eosin staining techniques and were later sent to immunohistochemistry to check for expression of antibodies p53 and Ki-67. Next, patients were compared in terms of age, histological grade, lymph node involvement, and tumor staging.

Immunohistochemistry tests were done from tissue samples previously fixated in 10% formalin, embedded in paraffin blocks, manually sliced at 3 microns using a rotational microtome, and mounted in organosilane. Each slide also contained immunohistochemically active control tissue to avoid false negative results. Slides were prepared as follows:

a. Preparation of histology cuts;
b. Deparaffination and hydration;
c. Antigenic recovery through irradiation in microwave oven;
d. Blocking endogenous peroxidase: slides were placed in a solution of 5% hydrogen peroxide and distilled water and then in a solution of 5% skim milk and phosphate buffer (PBS) for 40 minutes in a humid dark chamber; slides were then washed in water, distilled water, and placed in PBS for 5 minutes;
e. Incubation of primary antibody anti-Ki 67 and p53 (Dako);
f. Reaction of the avidin-biotin-streptavidin complex + peroxidase kit (Dako®): secondary antibody and marker;
g. Development;
h. Counterstaining

Negative result was defined as absence of stained nuclei or immunosuppression under 10% in tumor cells for markers p53 and Ki-67. Positive results were defined as 10% or more stained nuclei. This was based on criteria from our service, as there is no consensus in the literature as to the minimum thresholds to consider results as positive.

In terms of age, our patients were divided into two groups: (1) patients younger than 50 years (2) and patients 50 and older.

In terms of lymph node involvement, patients were divided into two groups: (1) positive neck lymph nodes for metastasis (pN1, pN2) and (2) negative lymph nodes for metastasis (pN0).

In terms of histological grade, epidermoid carcinomas were divided into two groups: (1) low grade, or well differentiated - keratinizing (grade I); and (2) high grade, or moderate to little differentiation (grades II and III).

In terms of disease staging (pT) cases were divided into two groups, according to the TNM System from the Union Internationale Contre Cancer (UICC): (1) cancer with local involvement (including pT1) and (2) cancer involving neighboring structures (including pT2, pT3, and pT4).

Statistical analysis was done using software program SPSS release 15.0. The chi-square test was used to compare categorical variables. The combined association between Ki-67 and p53 tumor immune expression and other analyzed variables (age, gender, lymph node involvement, histological grade, and disease staging [pT, pN]) was assessed through statistical analysis. Statistical significance was assigned when p<0.05.

This study was approved by the Research Ethics Committee at Universidade Luterana do Brasil under permit 2006-192H.

## RESULTS

This study included 30 patients (28 males and 2 females) histologically diagnosed with laryngeal epidermoid carcinoma. All subjects were submitted to total laryngectomy combined with neck clearance.

The results from the comparisons done between the expression of antibodies p53 and Ki-67, and prognostic factors (age, gender, lymph node involvement, histological grade, and disease staging) can be seen on Tables 1 and 2.

**Table 1:** Correlation between prognostic factors and the p53 gene expression in patients with laryngeal epidermoid carcinoma.

Prognostic factor	Positive p53	Negative p53	p
< 50 years	2 (50%)	2 (50%)	n.s.
> 50 years	16 (61,5%)	10 (38,5%)	n.s.
Positive Lymph nodes	9 (60%)	6 (40%)	n.s.
Negative lymph nodes	9 (60%)	6 (40%)	n.s.
Low grade	4 (40%)	6 (60%)	n.s.
High grade	14 (70%)	6 (30%)	n.s.
T1 staging	2 (50%)	2 (50%)	n.s.
T2 – T4 staging	16 (61,5%)	10 (38,5%)	n.s.

**Table 2:** Correlation between prognostic factors and Ki-67 antibody expression in patients with SCC.

Prognostic factor	Positive ki-67	Negative ki-67	p
< 50 years	2 (50%)	2 (50%)	n.s.
> 50 years	21 (80,8%)	5 (19,2%)	0,032
Positive lymph nodes	11 (73,3%)	4 (26,7%)	n.s.
Negative lymph nodes	12 (80%)	3 (20%)	n.s.
Low grade	8 (80%)	2 (20%)	n.s.
High grade	15 (75%)	5 (35%)	n.s.
T1 staging	4 (100%)	0 (0%)	n.s.
T2 – T4 staging	19 (73,1%)	7 (26,9%)	n.s.

Patient age ranged between 21 and 80 years (mean 56.2 years). In the group aged 50 and more, significantly higher levels of immune expression were seen for antibody Ki-67 (p = 0.032).

As for the other prognostic factors (lymph node involvement, histological grade, and disease staging), there was a trend towards increased p53 and Ki-67 immune expression in cases of higher histological grade and advanced disease, but such difference failed to achieve statistical significance (p>0.05).

## DISCUSSION

A lot has been found about prognostic markers for laryngeal carcinoma within the last few years. Nonetheless, reports of statistical correlations between p53 and Ki-67 expression and laryngeal cancer prognosis are yet inconsistently found in the literature.

The thirty patients in our study were aged between 21 and 80 years (mean 56.2 years). The groups were divided between subjects aged up to 50 and patients older than 50, and they were compared for cell markers p53 and Ki-67. The younger group had the same number of positive and negative cases, while in the older group the number of positive cases was higher for both markers (61.5% for p53 and 80.8% for Ki-67). These results indicate that older patients have higher tumor proliferation rate and thus more aggressive disease.

The association between p53 and lymph node metastasis was not proven, showing the equilibrium between marker expression and lymph node involvement (60% of the tumors were positive for p53, for both metastatic and non-metastatic disease). It failed to reveal statistical significance between groups.

Calli et al.^12^ investigated p53 and Ki-67 expression through immunohistochemistry in 37 patients diagnosed with laryngeal epidermoid carcinoma and found positive results in 83.8% of the cases for Ki-67 and 40% for p53, a statistically non-significant finding. Cruz et al.^13^ looked into 55 cases of oral cavity epidermoid carcinoma and reported positive results for p53 in 64% of the cases using a cut point of 25% of stained tumor cells. Kropveld et al.^14^ analyzed 25 patients and used immunohistochemistry and PCR to detect p53 mutations. They found association between p53 mutation and carcinoma in 100% of the cases using both methods and in 96% of the cases using immunohistochemistry alone, using however a cut point of 50% of stained tumor cells. Rozemuller et al.15 looked at 50 carcinoma patients and used PCR to associate head and neck epidermoid carcinoma to p53 expression in 95% of the cases.

Our findings are not fully in agreement with the literature. This is probably due to the fact that the authors did not choose only tumors in the larynx, but in the whole head and neck region. Bosch et al.^16^ reported that the prevalence of p53 alterations (mutation, expression, and expression loss) is significantly higher in hypopharyngeal tumors than in other sites.

Antibody p53 was positive in 50% of the cases in our study. Seventy percent of them were high histological grade tumors, and 60% had neck metastasis, possibly indicating that p53 immunohistochemical expression in epidermoid carcinoma patients is related to poor prognosis (high histological grade and regional metastasis).

Similarly to our study, Luo et al.17 investigated p53 expression through immunohistochemistry tests in 76 patients and failed to find statistically significant correlations between gender, age, and disease staging (pT), while associations with histological grades (I, II, and III) were statistically significant. From these findings the authors suggest that p53 expression present in early carcinomas may have important prognostic value. Another study supportive of this idea is the one published by Zhou et al.^18^, as p53 expression was found in 0%, 31%, and 52% of cases of epithelial hyperplasia without atypia, epithelial hyperplasia with atypia, and laryngeal epidermoid carcinoma respectively. This same study reports p53 expression rates of 62%, 76%, and 15% in well-differentiated carcinomas, moderately differentiated carcinomas, and poorly differentiated carcinomas respectively. The authors concluded that p53 expression is part of tumor pathogenesis and growth.

Seventy-six percent of the epidermoid carcinomas in our study presented immunohistochemical expression for Ki-67. Ki-67 expression was evident in 73.3% of the patients with metastatic lymph nodes and in 75% of high grade tumors. This suggests that Ki-67 may be related to poor prognosis factors (regional lymph node metastasis and high histological grade) in laryngeal epidermoid carcinoma cases (p>0.05). Liu et al.19 looked at 80 preoperative biopsies of patients histologically diagnosed with head and neck epidermoid carcinoma through immunohistochemistry for Ki-67 and correlated these findings to postoperative specimens, concluding that Ki-67 has a statistically significant predictive value to discern metastatic and non-metastatic carcinomas. Sun et al.^20^ analyzed 32 cases of laryngeal epidermoid carcinoma and correlated their findings to Ki-67, T disease stage, and presence of metastatic lymph nodes to conclude that Ki-67 is statistically related to present tumor and poor prognosis.

The literature also support the hypothesis that Ki-67 is associated with worse progress of laryngeal epidermoid carcinomas. Mirza et al.^21^ studied 80 patients histologically diagnosed with laryngeal mucosal dysplasia and performed immunohistochemistry tests for Ki-67. Ki-67 expression was categorized in terms of intensity in a scale from 0 to 4. The patients were followed and twenty of them evolved to malignant carcinoma. The authors concluded that Ki-67 at maximal expression levels (scores 3 and 4) was highly specific (80%) for laryngeal epidermoid carcinoma.

In terms of disease staging, p53 expression in early tumors was found in 50% of the cases, while Ki-67 expression was seen in 100% of the cases categorized as pT1 (n=4). In advanced disease ranging between stages pT2 and pT4 (n=26), p53 and Ki-67 expression was seen in 61.5% and 73.1% of the cases respectively. There was no statistically significant correlation between laryngeal carcinoma stage and immunohistochemical expression of the two analyzed antibodies.

## CONCLUSION

Except for the group aged 50 and more, in which Ki-67 expression was significantly higher, no statistically significant differences were found between p53 and Ki-67 expression and laryngeal epidermoid carcinoma.

The other prognostic factors (histological grade, lymph node involvement, and disease staging) require more research to thus increase the number of cases and studies based on DNA sequencing to verify the presence of mutations.
